# Efficacy, Safety, and Maintenance Use of Topical Bexarotene in Early‐Stage Mycosis Fungoides: A Single‐Center Retrospective Real‐World Study

**DOI:** 10.1111/1346-8138.70431

**Published:** 2026-07-27

**Authors:** Fatich Memet, Dilek Bayramgürler, Abdullah Demirbaş, Evren Odyakmaz Demirsoy, Aysun Şikar Aktürk, Nilgün Sayman, Rebiay Kıran

**Affiliations:** ^1^ Department of Dermatology and Venereology Kocaeli University Faculty of Medicine Kocaeli Türkiye

**Keywords:** cutaneous T‐cell lymphoma, maintenance therapy, mycosis fungoides, relapse, skin‐directed therapy, topical bexarotene

## Abstract

Topical bexarotene 1% gel is an approved skin‐directed treatment for refractory or persistent stage IA‐IB cutaneous T‐cell lymphoma, but real‐world evidence in mycosis fungoides (MF), particularly regarding maintenance use after complete response, remains limited. We retrospectively reviewed 56 patients with histopathologically and immunohistochemically confirmed, previously treated stage IA‐IB MF who received topical bexarotene between 2009 and 2022. Clinical response, post‐treatment biopsy findings, adverse events, maintenance therapy, and relapse outcomes were analyzed. The cohort included 31 women and 25 men; 42 patients (75.0%) had stage IA and 14 (25.0%) had stage IB disease. Complete response (CR) was achieved in 20 patients (35.7%) and partial response in 15 (26.8%), yielding an overall response rate of 62.5%. Overall response was higher in stage IA than in stage IB disease (73.8% vs. 28.6%; *p* = 0.003) and in T1a than in T2a disease (77.1% vs. 36.4%; *p* = 0.044). All biopsied complete responders showed no MF‐compatible histology. Adverse events were exclusively local and manageable. Among 18 evaluable complete responders, relapse occurred in 15 (83.3%). Median time to relapse was longer with maintenance topical bexarotene than without maintenance (45 vs. 30 weeks; *p* = 0.039). Topical bexarotene appears effective and well tolerated in previously treated stage IA‐IB MF, with greatest benefit in limited patch‐stage disease. Maintenance use after CR may delay relapse, but this exploratory finding requires prospective validation.

## 1 Introduction

Mycosis fungoides (MF) is the most frequent subtype of primary cutaneous T‐cell lymphoma and typically follows an indolent but chronically relapsing course. In early‐stage disease, malignant T‐cell infiltration remains primarily confined to the skin, and treatment aims to provide durable disease control with minimal toxicity rather than definitive cure. Current staging relies on the revised International Society for Cutaneous Lymphomas/European Organization for Research and Treatment of Cancer TNMB classification [1].

Skin‐directed therapies remain the cornerstone of early‐stage MF management. Topical corticosteroids, phototherapy, topical mechlorethamine/chlormethine, localized radiotherapy, and topical retinoids are commonly used sequentially or in combination according to disease extent, lesion morphology, availability, tolerability, and previous response [2, 3, 4, 5, 6, 7].

Topical bexarotene 1% gel is a selective retinoid X receptor agonist indicated for refractory or persistent stage IA‐IB cutaneous T‐cell lymphoma after other therapies or intolerance to prior therapies [8]. The pivotal evidence base includes the phase I/II trial by Breneman et al. and the multicenter phase III trial by Heald et al. [9, 10] Despite regulatory approval and long‐standing clinical use, real‐world data remain limited, and the potential role of intermittent maintenance after complete response (CR) is not well characterized.

We report a 13‐year single‐center real‐world experience with topical bexarotene in previously treated stage IA‐IB MF. We evaluated clinical response, safety, post‐treatment histopathological findings, and relapse outcomes, with particular emphasis on whether maintenance treatment after CR was associated with longer remission duration.

## 2 Materials and Methods

### 2.1 Study Design and Ethics

This retrospective single‐center observational study was conducted at the Cutaneous Lymphomas Outpatient Clinic, Department of Dermatology and Venereology, Kocaeli University Faculty of Medicine Hospital. The study period covered patients treated between January 2009 and December 2022. Reporting followed the principles of transparent observational study reporting, including explicit eligibility criteria, patient flow, outcome definitions, and limitations related to retrospective data capture [11].

The study was approved by the local ethics committee of Kocaeli University Faculty of Medicine (November 21, 2024; Project No: 2024/464) and was conducted in accordance with the Declaration of Helsinki. Because of the retrospective design, informed consent requirements were handled according to institutional ethics committee procedures.

### 2.2 Patient Selection and Baseline Assessment

Records of 81 patients with MF who had received topical bexarotene were screened. Patients were eligible if they had histopathologically and immunohistochemically confirmed MF, stage IA or IB disease according to ISCL/EORTC staging, and sufficient clinical follow‐up to assess response. All included patients had received at least one prior skin‐directed therapy without achieving CR or had relapsed after CR to previous treatment.

Patients were excluded if follow‐up was irregular or insufficient for response assessment (*n* = 22), concomitant acitretin use prevented attribution of response to topical bexarotene (*n* = 2), or treatment was stopped very early because of intolerance before meaningful response assessment (*n* = 1). The final analytic cohort included 56 patients.

Disease stage was assigned using the revised ISCL/EORTC TNMB classification [1]. Skin classification was recorded as T1a (< 10% body surface area [BSA], patches only), T1b (< 10% BSA, plaques with or without patches), T2a (≥ 10% BSA, patches only), or T2b (≥ 10% BSA, plaques with or without patches). Stage IIA or higher disease was not included.

Nodal status was evaluated by clinical examination and superficial lymph node ultrasonography when available. Peripheral blood involvement was screened by peripheral blood smear in all patients and by flow cytometry in a subset according to clinical indication and historical availability. Baseline lactate dehydrogenase and beta‐2 microglobulin were recorded when available.

### 2.3 Treatment, Response Assessment, and Relapse Definition

Topical bexarotene 1% gel was applied to clinically involved skin. Application frequency was individualized and titrated from once daily up to four times daily according to tolerability, local irritation, lesion burden, and physician judgment. Dose reduction, temporary interruption, emollients, or short courses of topical corticosteroids were used to manage local irritation when necessary.

Consistent with the prescribing information, treatment was generally initiated at a low frequency and escalated according to local tolerability. The labeled regimen recommends initial application once every other day during the first week, followed by weekly escalation to once daily, twice daily, three times daily, and then four times daily as tolerated. In routine practice, escalation was not determined solely by plaque thickness; lesion burden, lesion morphology, anatomic site, local irritation, and patient tolerance were also considered. Thus, thicker or plaque‐predominant lesions could prompt careful upward titration, but the retrospective records did not allow a reliable lesion‐level analysis of dosing according to plaque thickness.

Response was assessed retrospectively from dermatology records and clinical documentation. CR was defined as complete clinical clearance of MF‐compatible lesions. Partial response (PR) was defined as at least 50% reduction in lesion burden. Stable disease (SD) was defined as persistence without sufficient improvement for PR and without progression. Progressive disease (PD) was defined as worsening, new clinically significant MF lesions, or progression requiring treatment change. Because this was retrospective, response was based on physician global assessment and lesion clearance documented in routine practice rather than prospectively captured modified Severity Weighted Assessment Tool scores [12].

Post‐treatment skin biopsy was performed at the discretion of the treating physician, particularly when residual disease was clinically uncertain or confirmation of clearance was considered useful. Biopsy findings were categorized as no MF‐compatible histology or persistent MF‐compatible histology.

Maintenance topical bexarotene was defined as intermittent continued treatment after CR, usually one to three applications per week. The decision to use maintenance treatment was not randomized and reflected physician judgment, tolerability, patient preference, and perceived relapse risk.

Relapse was defined as reappearance of clinically compatible MF lesions after CR during follow‐up, leading to continued, reinitiated, or intensified treatment. Patients without relapse were considered relapse‐free at the last available follow‐up. Because patient‐level relapse and censoring times were not available in the aggregate extraction used for this manuscript, relapse outcomes are summarized descriptively rather than as formal Kaplan–Meier estimates.

### 2.4 Statistical Analysis

Continuous variables were summarized as mean ± standard deviation or median and interquartile range, as appropriate. Categorical variables were summarized as number and percentage. Between‐group comparisons used chi‐square, Fisher exact, or Mann–Whitney *U* tests according to variable type and distribution. A two‐sided *p* value < 0.05 was considered statistically significant. Subgroup analyses with small cell counts were interpreted in the context of clinical plausibility and sample size limitations.

## 3 Results

### 3.1 Patient Characteristics and Treatment Exposure

Among 81 screened patients, 25 were excluded, leaving 56 patients in the final analytic cohort (Figure 1; Table 1). The cohort included 31 women (55.4%) and 25 men (44.6%). Mean age at diagnosis was 44.4 ± 14.0 years, and mean diagnostic delay was 50.1 ± 43.4 months. Classic MF was the predominant clinical type (89.3%). By TNMB stage, 42 patients (75.0%) had stage IA and 14 (25.0%) had stage IB disease; no patient with stage IIA or higher disease was included.

**FIGURE 1:**
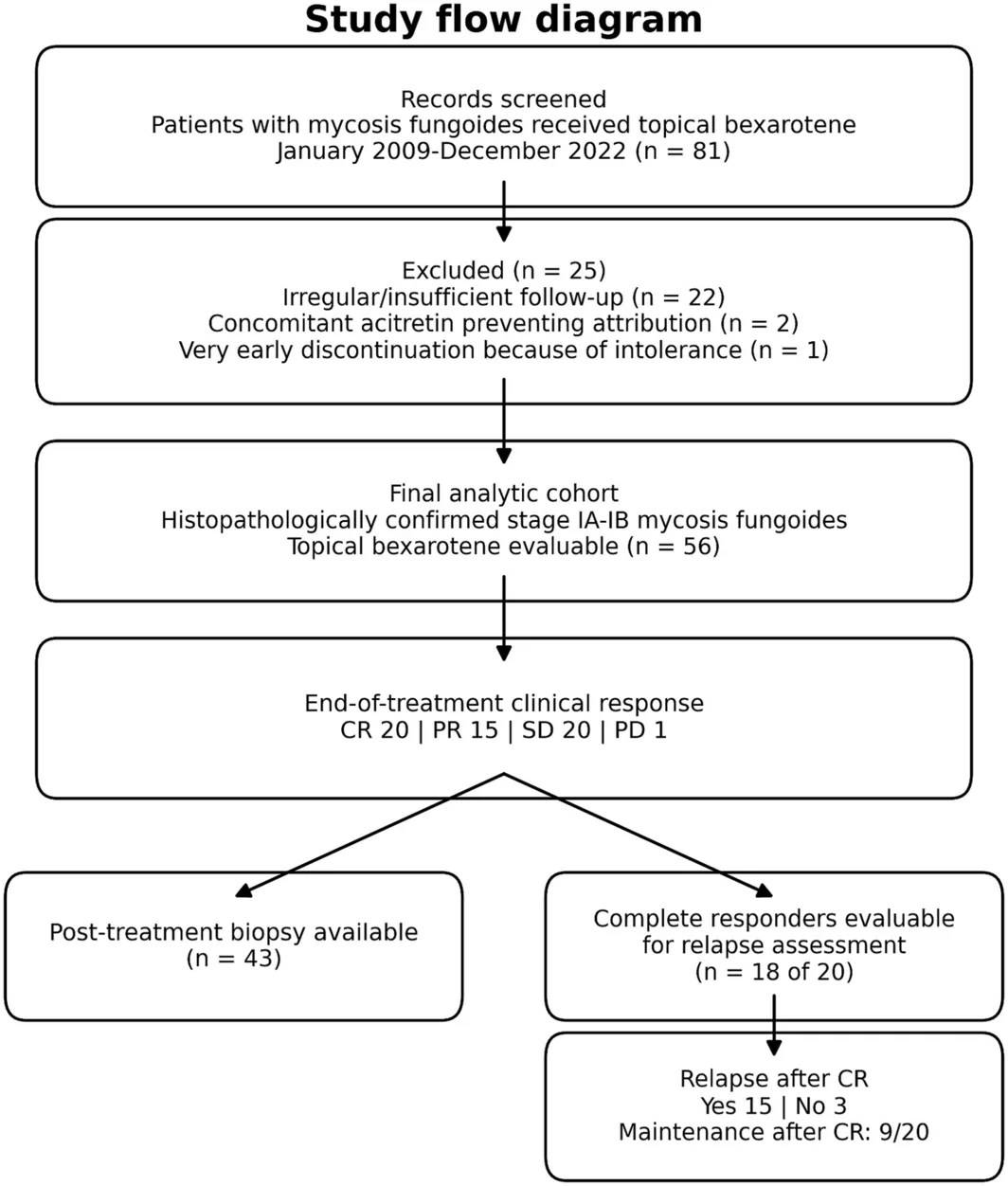
Patient flow diagram showing screened records, exclusion reasons, and the final analytic cohort.

**TABLE 1:** Baseline characteristics, previous therapies, and topical bexarotene treatment exposure (*n* = 56).

Domain	Parameter	Value
Baseline characteristics	Sex, female/male	31 (55.4%) / 25 (44.6%)
Baseline characteristics	Age at diagnosis, mean ± SD, years	44.4 ± 14.0
Baseline characteristics	Diagnostic delay, mean ± SD, months	50.1 ± 43.4
Baseline characteristics	Classic MF	50 (89.3%)
Baseline characteristics	Poikilodermatous MF	3 (5.4%)
Baseline characteristics	Pigmented purpuric dermatosis‐like MF	2 (3.6%)
Baseline characteristics	Hypopigmented MF	1 (1.8%)
Baseline characteristics	Stage IA	42 (75.0%)
Baseline characteristics	Stage IB	14 (25.0%)
Baseline characteristics	T1a, patches < 10% BSA	35 (62.5%)
Baseline characteristics	T1b, plaques < 10% BSA	7 (12.5%)
Baseline characteristics	T2a, patches ≥ 10% BSA	11 (19.6%)
Baseline characteristics	T2b, plaques ≥ 10% BSA	3 (5.4%)
Baseline characteristics	Elevated LDH among tested patients	6/36 (16.7%)
Baseline characteristics	Elevated beta‐2 microglobulin among tested patients	8/23 (34.8%)
Baseline characteristics	Normal peripheral blood smear	56 (100%)
Previous therapies	Topical corticosteroids	52 (92.9%)
Previous therapies	Narrowband ultraviolet B	25 (44.6%)
Previous therapies	Psoralen plus ultraviolet A	23 (41.1%)
Previous therapies	Retinoid‐assisted phototherapy	19 (33.9%)
Previous therapies	Topical tazarotene	4 (7.1%)
Previous therapies	Acitretin	5 (8.9%)
Previous therapies	Interferon‐alpha	4 (7.1%)
Previous therapies	Systemic bexarotene	2 (3.6%)
Previous therapies	Number of prior treatments, median (range)	2 (1–5)
Indication	Failure to achieve CR after previous therapy	54 (96.4%)
Indication	Relapse after previous CR	2 (3.6%)
Treatment exposure	Treatment duration, mean ± SD, weeks	34.89 ± 18.77
Treatment exposure	Treatment duration, range, weeks	14–106
Treatment exposure	Treatment duration in men vs. women, mean ± SD, weeks	41.16 ± 22.59 vs. 29.84 ± 13.33; *p* = 0.028
Maximum daily frequency	Once daily	2 (3.6%)
Maximum daily frequency	Twice daily	19 (33.9%)
Maximum daily frequency	Three times daily	15 (26.8%)
Maximum daily frequency	Four times daily	20 (35.7%)

*Note:* Some patients received more than one prior therapy; percentages therefore do not sum to 100%. Maximum daily frequency reflects the highest tolerated frequency during treatment.

Abbreviations: BSA, body surface area; CR, complete response; LDH, lactate dehydrogenase; MF, mycosis fungoides; PUVA, psoralen plus ultraviolet A; SD, standard deviation.

No folliculotropic MF cases were present in the analytic cohort; therefore, ORR could not be compared between folliculotropic and non‐folliculotropic subtypes.

All patients had received at least one prior skin‐directed therapy before topical bexarotene. The median number of prior treatments was 2 (range, 1–5). Topical corticosteroids were the most common previous therapy (92.9%), followed by narrowband ultraviolet B (44.6%) and psoralen plus ultraviolet A (41.1%). Topical bexarotene was initiated because of failure to achieve CR in 54 patients (96.4%) and because of relapse after previous CR in 2 (3.6%). Treatment duration ranged from 14 to 106 weeks, with a mean of 34.89 ± 18.77 weeks. Previous therapies and treatment exposure are summarized in Table 1.

The mean maximum tolerated application frequency was 2.95 ± 0.92 applications/day, with a median of 3 applications/day (interquartile range, 2–4). Overall, 54 of 56 patients (96.4%) reached a maximum frequency of at least twice daily. By disease stage, maximum once‐, twice‐, three‐, and four‐times‐daily frequencies were observed in 2, 14, 12, and 14 patients with stage IA disease and in 0, 5, 3, and 6 patients with stage IB disease, respectively (Table S4). Time‐weighted average application frequency could not be reliably calculated because dosing frequency often changed during treatment according to tolerability and local reactions; therefore, maximum tolerated frequency is reported.

### 3.2 Clinical Response

Overall, CR was achieved in 20 patients (35.7%) and PR in 15 (26.8%), yielding an overall response rate (ORR) of 62.5%. SD was observed in 20 patients (35.7%), and PD occurred in 1 (1.8%) (Figure 2A; Table 2).

**FIGURE 2:**
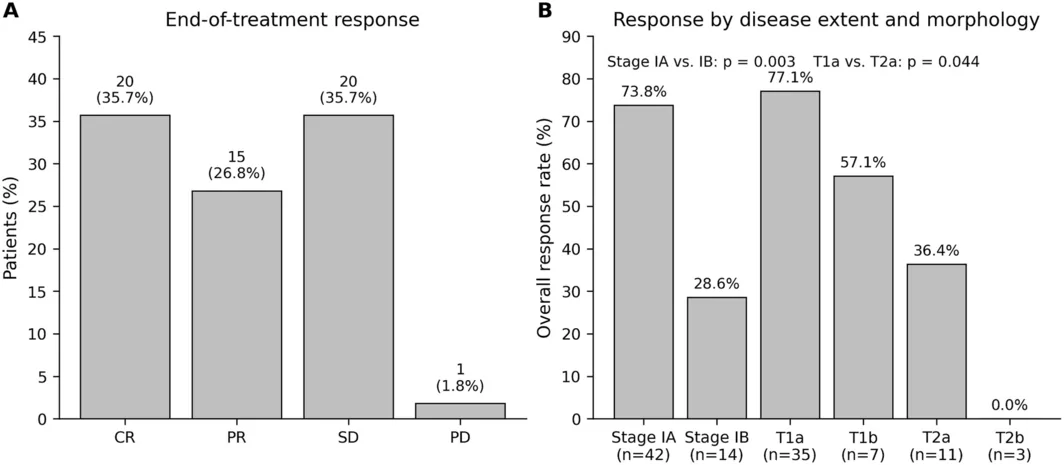
Clinical response after topical bexarotene. (A) Distribution of complete response, partial response, stable disease, and progressive disease. (B) Overall response rate according to disease stage and T‐substage.

**TABLE 2:** Treatment response according to disease stage, sex, and T‐substage.

Variable	Subgroup	*n*	CR	PR	ORR	SD	PD	Key comparison
Overall	All patients	56	20 (35.7%)	15 (26.8%)	35 (62.5%)	20 (35.7%)	1 (1.8%)	—
Stage	IA	42	18 (42.9%)	13 (31.0%)	31 (73.8%)	11 (26.2%)	0 (0%)	ORR *p* = 0.003; SD *p* = 0.022
Stage	IB	14	2 (14.3%)	2 (14.3%)	4 (28.6%)	9 (64.3%)	1 (7.1%)	ORR *p* = 0.003; SD *p* = 0.022
Sex	Male	25	13 (52.0%)	3 (12.0%)	16 (64.0%)	9 (36.0%)	0 (0%)	ORR *p* = 1.000
Sex	Female	31	7 (22.6%)	12 (38.7%)	19 (61.3%)	11 (35.5%)	1 (3.2%)	CR *p* = 0.028; PR *p* = 0.034
T‐substage	T1a	35	15 (42.9%)	12 (34.3%)	27 (77.1%)	8 (22.9%)	0 (0%)	T1a vs. T2a ORR *p* = 0.044
T‐substage	T1b	7	3 (42.9%)	1 (14.3%)	4 (57.1%)	3 (42.9%)	0 (0%)	NS vs. T1a
T‐substage	T2a	11	2 (18.2%)	2 (18.2%)	4 (36.4%)	6 (54.5%)	1 (9.1%)	T1 overall vs. T2 overall ORR *p* = 0.003
T‐substage	T2b	3	0 (0%)	0 (0%)	0 (0%)	3 (100%)	0 (0%)	Exploratory

*Note:* Small T1b and T2b subgroups should be interpreted cautiously.

Abbreviations: CR, complete response; NS, not significant; ORR, overall response rate; PD, progressive disease; PR, partial response; SD, stable disease.

Response differed significantly by disease extent. ORR was higher in stage IA than in stage IB disease (73.8% vs. 28.6%; *p* = 0.003). SD was more frequent in stage IB disease (64.3% vs. 26.2%; *p* = 0.022), and the single case of PD occurred in stage IB disease. When response was evaluated by T‐substage, ORR was highest in T1a disease (77.1%) and lowest in T2b disease (0%). ORR was significantly higher in T1a than in T2a disease (77.1% vs. 36.4%; *p* = 0.044) (Figure 2B; Table 2). Comparisons involving the small T1b and T2b subgroups were interpreted cautiously.

CR was more frequent among men than women (52.0% vs. 22.6%; *p* = 0.028), whereas PR was more frequent among women (38.7% vs. 12.0%; *p* = 0.034). Overall ORR did not differ significantly by sex (64.0% in men vs. 61.3% in women; *p* = 1.000). Men had a longer mean treatment duration than women (41.16 ± 22.59 vs. 29.84 ± 13.33 weeks; *p* = 0.028).

### 3.3 Clinicopathological Concordance and Safety

Post‐treatment biopsies were available in 43 patients. Among 13 biopsied patients with clinical CR, all showed no histopathological evidence of MF. Among 12 biopsied patients with PR, 8 (66.7%) showed no residual MF‐compatible histology, whereas 4 (33.3%) had persistent MF findings. Among 18 biopsied patients with SD, 17 (94.4%) had persistent MF‐compatible histology (Table S1).

Adverse events were reported in 54 patients (96.4%) and were exclusively local cutaneous reactions at application sites. Erythema or irritation occurred in all patients with adverse events, burning or stinging in 12 (22.2%), and pruritus in 7 (13.0%). No systemic adverse events were recorded, and no patient required permanent treatment discontinuation because of adverse events (Table S1).

### 3.4 Maintenance Treatment and Relapse

Among the 20 patients who achieved CR, 9 (45.0%) received maintenance topical bexarotene one to three times weekly. Relapse assessment was available for 18 complete responders. Relapse occurred in 15 of 18 patients (83.3%). Among relapsed complete responders, observed median time to relapse was longer with maintenance treatment than without maintenance (45 weeks, interquartile range 39.5–107.5, vs. 30 weeks, interquartile range 18.0–50.5; *p* = 0.039) (Figure 3; Table S2). The three patients who remained relapse‐free had a mean follow‐up of 108.3 weeks.

**FIGURE 3:**
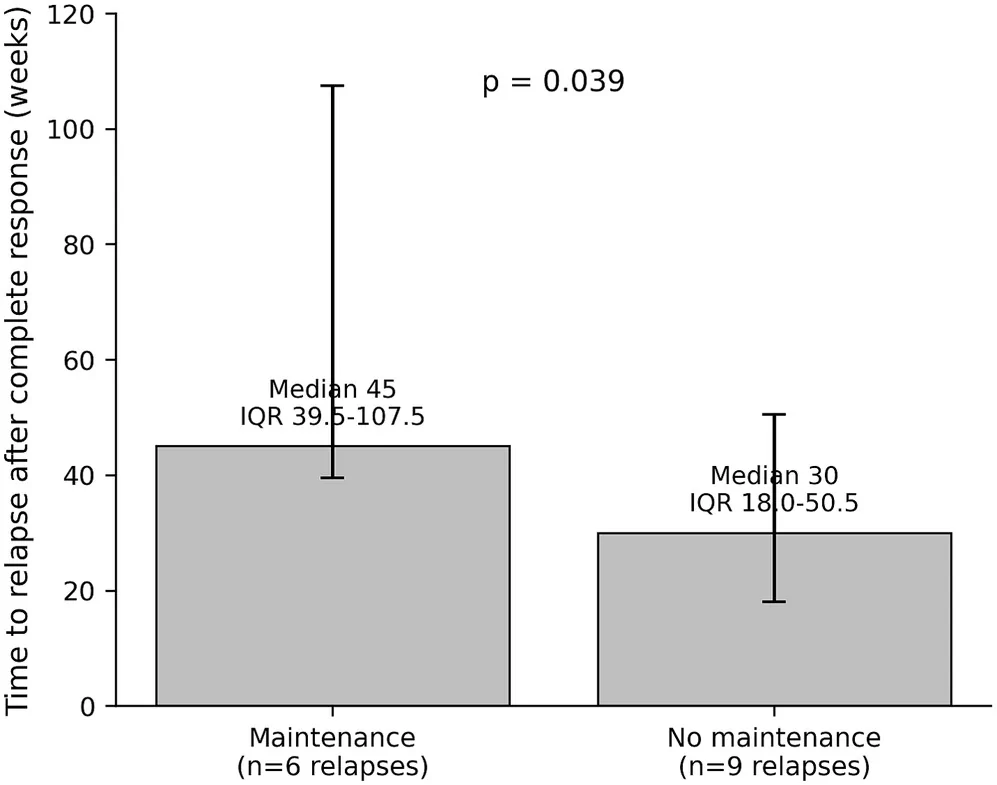
Maintenance topical bexarotene and observed time to relapse among relapsing complete responders. Bars show medians and error bars show interquartile ranges. The comparison is exploratory because maintenance was not randomized and patient‐level censoring data were unavailable.

## 4 Discussion

This 13‐year real‐world study suggests that topical bexarotene is an effective and well‐tolerated skin‐directed therapy for stage IA‐IB MF after failure of prior skin‐directed treatments. The ORR of 62.5% and CR rate of 35.7% are clinically meaningful in a cohort composed entirely of previously treated patients. Responses were concentrated in stage IA and T1a disease, reinforcing the practical principle that limited patch‐stage involvement is more likely to respond to topical therapy than more extensive disease.

The efficacy results are consistent with pivotal and comparative studies (Table S3). Breneman et al. [9] reported an ORR of approximately 63% and a CR rate of 21% in a phase I/II trial of topical bexarotene in cutaneous T‐cell lymphoma. Heald et al. [10] subsequently demonstrated clinical activity in refractory or persistent early‐stage disease, with response varying by assessment method. Cerman et al. [13] reported an ORR of 80% in the topical bexarotene arm of a comparative early‐stage MF study and longer remission after bexarotene than after clobetasol propionate. Their higher ORR may reflect differences in baseline population and treatment context, whereas all patients in the present cohort had previously failed at least one skin‐directed therapy.

From a practical therapeutic perspective, these data help position topical bexarotene as a useful option after inadequate response to more frequently used skin‐directed therapies. The response gradient across stage and T‐substage suggests that clinicians should consider disease burden and lesion morphology when selecting patients for this agent. Patients with limited patch‐stage disease may be the most appropriate candidates, whereas patients with more extensive or plaque‐dominant disease may require earlier combination approaches or transition to alternative skin‐directed or systemic strategies.

The role of topical bexarotene should also be considered alongside chlormethine/mechlorethamine gel, an established skin‐directed therapy for persistent or refractory early‐stage MF lesions. In the pivotal randomized Study 201, chlormethine gel achieved confirmed response rates of 58.5% by Composite Assessment of Index Lesion Severity and 46.9% by Severity‐Weighted Assessment Tool, with complete response rates of 13.8% and 6.9%, respectively [14]. Subsequent analyses reported accumulating responses with chlormethine gel and suggested that treatment‐associated dermatitis may coexist with clinical benefit in some patients [15, 16]. Real‐world evaluations have similarly supported its effectiveness and manageable local toxicity profile [17]. In this context, the ORR of 62.5% observed in the present previously treated cohort is broadly comparable with published chlormethine gel data, although direct comparison is limited by differences in study design, response instruments, stage distribution, prior treatment exposure, and the absence of prospectively captured mSWAT scores. Clinically, both agents appear most useful as lesion‐directed therapies in early‐stage disease; chlormethine gel has a direct cytotoxic mechanism and extensive trial support, whereas topical bexarotene offers a retinoid‐based alternative for patients with persistent or refractory lesions, particularly when local tolerability and patient preference favor this approach.

The stage‐specific findings are clinically important. ORR was 73.8% in stage IA but 28.6% in stage IB disease, and T1a disease showed a higher ORR than T2a disease. The lack of response in the T2b subgroup should be interpreted with caution because only three patients were included, but it is biologically plausible that thicker plaques and greater skin burden are less responsive to a local retinoid alone.

The observed sex‐related response pattern is noteworthy but should not be overinterpreted. Men had a higher CR rate, whereas women had a higher PR rate; however, overall ORR was similar between sexes. Men also had longer treatment duration, which may have increased the probability of complete clearance. Unmeasured differences in lesion morphology, distribution, adherence, or treatment tolerance may also have contributed.

The histopathological analysis provides additional clinical context. In biopsied patients, clinical CR was consistently associated with the absence of MF‐compatible histology, whereas SD was strongly associated with persistent MF histology. This does not prove universal pathological clearance because biopsies were not obtained in all patients and biopsy decisions were not standardized. Nevertheless, the findings support the clinical relevance of documented complete clearance and suggest that biopsy may be useful when residual disease is uncertain or when treatment discontinuation is being considered.

This point is relevant because early‐stage MF often leaves subtle erythema, pigmentation, or textural change after treatment, and these residual findings may not always represent active lymphoma. Conversely, clinically persistent lesions may retain histologic features of MF even when partial improvement is observed. In selected patients, post‐treatment biopsy may therefore support more rational decisions about stopping therapy, continuing treatment, or moving to another skin‐directed modality.

The maintenance analysis is the most clinically relevant exploratory aspect of this study. Intermittent maintenance topical bexarotene after CR was associated with longer observed time to relapse. This observation aligns with the broader concept that maintenance or prolonged skin‐directed therapy may help extend remission in a chronic relapsing disease such as MF, although evidence differs by modality and setting [18, 19, 20, 21, 22]. Because maintenance was not randomized, the number of complete responders was small and patient‐level censoring data were unavailable, this result should be interpreted as an exploratory association rather than evidence of a causal relapse‐preventive effect.

The safety profile was favorable. Although local irritation was common, it was manageable with moisturization, temporary dose adjustment, or short courses of topical corticosteroids and did not lead to permanent discontinuation. No systemic adverse events were recorded. This local toxicity profile is consistent with the pharmacologic and clinical profile of topical bexarotene, which is directed to cutaneous lesions and generally has limited systemic exposure when used appropriately [8, 9, 10].

Patient education is therefore central to successful use. Gradual titration, proactive emollient use, temporary interruption during irritation, and realistic counseling about the time required for response may improve adherence and prevent premature discontinuation in everyday routine practice, especially in patients who have already experienced treatment fatigue after multiple previous skin‐directed therapies.

This study has limitations. The retrospective single‐center design introduces selection bias and limits generalizability. Treatment frequency, duration, maintenance use, and biopsy decisions were individualized rather than protocol‐driven. Response was assessed by physician global assessment and lesion clearance rather than prospectively captured mSWAT scores. The absence of a concurrent control group prevents direct comparison with other skin‐directed therapies. The maintenance analysis is particularly limited by non‐randomized allocation, small sample size, and unavailable patient‐level censoring structure. In addition, routine records captured maximum tolerated application frequency rather than time‐weighted average exposure, and lesion‐specific escalation according to plaque thickness could not be analyzed.

The study also has strengths that are relevant for a concise clinical report. The cohort reflects routine tertiary‐care practice over a long treatment period, all included patients were previously treated, and the analysis integrates clinical response, post‐treatment biopsy findings, local tolerability, and relapse‐related outcomes. This combination provides a clinically useful view of how topical bexarotene performs beyond pivotal trial settings and identifies maintenance treatment as a question that merits prospective evaluation rather than as a definitive recommendation.

In conclusion, topical bexarotene appears to be an effective and well‐tolerated second‐line skin‐directed therapy for previously treated stage IA‐IB MF, with greatest benefit in stage IA and T1a disease. Clinical CR was supported by post‐treatment histopathological findings in biopsied patients. Intermittent maintenance after CR may delay relapse, but this finding should be regarded as hypothesis‐generating. Prospective or multicenter patient‐level studies are needed to define optimal maintenance schedules, treatment duration, relapse‐prevention benefit, and patient selection criteria.

## Funding

The authors have nothing to report.

## Conflicts of Interest

The authors declare no conflicts of interest.

## Supporting information


**Table S1:** Post‐treatment biopsy findings and safety outcomes.
**Table S2:** Maintenance topical bexarotene and relapse outcomes among evaluable complete responders.
**Table S3:** Contextual comparison with key topical bexarotene studies.
**Table S4:** Maximum tolerated daily application frequency according to disease stage.

## Data Availability

The data underlying this study are available from the corresponding author upon reasonable request and subject to institutional and ethical restrictions.
