# Supplementary material for: Efficacy, Safety, and Maintenance Use of Topical Bexarotene in Early‐Stage Mycosis Fungoides: A Single‐Center Retrospective Real‐World Study
Source: J Dermatol. 2026 Jul 27;53(10):1457–64. doi: 10.1111/1346-8138.70431 (PMC13646853; doi:10.1111/1346-8138.70431)
Supplement: Supplementary file 1 [file JDE-53-1457-s001.docx]

**Supplementary Material**

Efficacy, Safety, and Maintenance Use of Topical Bexarotene in Early-Stage Mycosis Fungoides: A Single-Center Retrospective Real-World Study

**Supplementary Table 1. Post-treatment biopsy findings and safety outcomes.**

| **Domain** | **Clinical category or event** | **n** | **No MF histology** | **MF present or event** | **p value** |
| --- | --- | --- | --- | --- | --- |
| Post-treatment biopsy | CR | 13 | 13 (100%) | 0 (0%) | <0.001 |
| Post-treatment biopsy | PR | 12 | 8 (66.7%) | 4 (33.3%) | 0.320 |
| Post-treatment biopsy | SD | 18 | 1 (5.6%) | 17 (94.4%) | <0.001 |
| Post-treatment biopsy | Total | 43 | 22 (51.2%) | 21 (48.8%) | - |
| Adverse events | Any local adverse event | 54 | - | 54 (96.4%) | - |
| Adverse events | Erythema/irritation | 54 | - | 54 (100% of AEs) | - |
| Adverse events | Burning/stinging | 54 | - | 12 (22.2%) | - |
| Adverse events | Pruritus | 54 | - | 7 (13.0%) | - |
| Adverse events | Permanent discontinuation due to AE | 56 | - | 0 (0%) | - |
| Adverse events | Systemic adverse event | 56 | - | 0 (0%) | - |

AE, adverse event; CR, complete response; MF, mycosis fungoides; PR, partial response; SD, stable disease. Biopsies were performed according to physician judgment and were not available for all patients.

**Supplementary Table 2. Maintenance topical bexarotene and relapse outcomes among evaluable complete responders.**

| **Maintenance topical bexarotene** | **Evaluable CR patients, n** | **Relapsed** | **Relapse-free at last follow-up** | **Median time to relapse among relapsed patients, weeks (IQR)** | **p value** |
| --- | --- | --- | --- | --- | --- |
| Administered | 9 | 6 (66.7%) | 3 (33.3%) | 45 (39.5-107.5) | 0.039 |
| Not administered | 9 | 9 (100%) | 0 (0%) | 30 (18.0-50.5) |  |

CR, complete response; IQR, interquartile range. Two patients with CR were not evaluable for relapse. The comparison is descriptive/exploratory because maintenance allocation was not randomized and patient-level censoring data were unavailable for formal time-to-event modeling.

**Supplementary Table 3. Contextual comparison with key topical bexarotene studies.**

| **Study** | **Design** | **Population** | **ORR** | **CR** | **Key relevance** |
| --- | --- | --- | --- | --- | --- |
| Breneman et al., 2002 | Phase I/II dose-escalation | 67 adults with early-stage CTCL | 63% | 21% | Foundational topical bexarotene efficacy trial; median response approximately 20 weeks. |
| Heald et al., 2003 | Multicenter phase III | 50 patients with refractory/persistent stage IA-IIA CTCL | 44-54% depending on assessment method | 2% by physician global assessment | Confirmed efficacy and tolerability in refractory/persistent early-stage CTCL. |
| Cerman et al., 2025 | Retrospective comparative study | 20 patients treated with topical bexarotene; stage IA-IB MF | 80% | 55% | Higher ORR likely reflects differences in baseline population and treatment context; remission was longer with bexarotene than with clobetasol. |
| Current study | Single-center retrospective real-world cohort | 56 previously treated patients with stage IA-IB MF | 62.5% | 35.7% | Adds post-treatment clinicopathological concordance and exploratory maintenance-relapse analysis. |

CR, complete response; CTCL, cutaneous T-cell lymphoma; MF, mycosis fungoides; ORR, overall response rate.

**Supplementary Table 4. Maximum tolerated daily application frequency according to disease stage.**

| **Maximum tolerated daily application frequency** | **Overall (n = 56)** | **Stage IA (n = 42)** | **Stage IB (n = 14)** |
| --- | --- | --- | --- |
| Once daily | 2 (3.6%) | 2 (4.8%) | 0 (0%) |
| Twice daily | 19 (33.9%) | 14 (33.3%) | 5 (35.7%) |
| Three times daily | 15 (26.8%) | 12 (28.6%) | 3 (21.4%) |
| Four times daily | 20 (35.7%) | 14 (33.3%) | 6 (42.9%) |
| At least twice daily | 54 (96.4%) | 40 (95.2%) | 14 (100%) |

This table reports the highest tolerated application frequency during treatment rather than time-weighted average exposure. Application frequency was individualized according to lesion burden, lesion morphology, anatomic site, local irritation, and patient tolerance.
